# Spatial heterogeneities of human-mediated dispersal vectors accelerate the range expansion of invaders with source–destination-mediated dispersal

**DOI:** 10.1038/s41598-020-78633-3

**Published:** 2020-12-08

**Authors:** Daisuke Takahashi, Young-Seuk Park

**Affiliations:** Department of Biology, Kyung Hee University, Seoul, 02447 Republic of Korea

**Keywords:** Ecology, Invasive species

## Abstract

Rapid range expansions of invasive species are a major threat to ecosystems. Understanding how invasive species increase their habitat ranges and how environmental factors, including intensity of human activities, influence dispersal processes is an important issue in invasion biology, especially for invasive species management. We have investigated how spatially heterogeneous factors influence range expansion of an invasive species by focusing on long-distance dispersal, which is frequently assisted by human activities. We have developed models varying two underlying processes of a dispersal event. These events are described by source and destination functions that determine spatial variations in dispersal frequency and the probability of being a dispersal destination. Using these models, we investigated how spatially heterogeneous long-distance dispersal influences range expansion. We found that: (1) spatial variations in the destination function slow down late population dynamics, (2) spatial variations in the source function increase the stochasticity of early population dynamics, and (3) the speed of early population dynamics changes when both the source and the destination functions are spatially heterogeneous and positively correlated. These results suggest an importance of spatial heterogeneity factors in controlling long-distance dispersal when predicting the future spread of invasive species.

## Introduction

Habitat invasion by alien species introduced by human activity is currently a major global issue in ecosystem management. While only a small fraction of alien species appear to be invasive^1^, they can exert catastrophic effects on ecosystems and human society^1–4^. Invasive species mostly share ability to establish colonies far away from a parent colony^5^. Therefore, their dispersals are a longstanding subject of empirical and theoretical studies^6^.

Dispersal is an important factor in determining a species’ spatial distribution. The dispersal is often characterized by a dispersal kernel, which is a probability distribution of dispersal success by distance. Dispersal with exponentially bounded tail of the dispersal kernel implies finite range expansion velocities^7^; this type of dispersal is called as short-distance dispersal. Otherwise, e.g., with a power-law dispersal kernel corresponding to Lévy flight (which is often applied to describe animal dispersal^8^ and human traveling^9,10^), the range expansion velocity is longer finite in general^7^, and emergent spatial distribution of the species scatters over entire area^11^. This type of dispersal is called long-distance dispersal, and has been applied to model intermittent jumps from the existing range to remote areas.

Dispersal of organisms consists of several different processes, e.g., processes mediated by species’ inherent dispersal ability and human transfer, and those processes may have different shapes of their dispersal kernels. Among invasive species often human activities mediate their dispersal that allow rapid range expansions^12^. Moreover, meta-analyses suggest humans as the major vector spreading invasive species ^5,13^. Distances of human traveling often show power-law distribution^9,10^; therefore, this human-mediated dispersal can give an ability of long-distance dispersal to invader species, which may otherwise spread slowly by their limited inherent dispersal abilities. Therefore, invasive species’ range expansion is better modeled as a stratified dispersal that considers more than one processes is involved in the spread of the invasive species^12^.

Alongside human-mediated long-distance dispersal, empirical studies have shown positive correlations between local human activities and the frequency of observation of invasive species^14–16^. Numerous studies have concluded that invasive species are more likely to be established in areas of high human activity. Gravity models (a common approach to describe spatial expansion of invader species) often determine dispersal destination by levels of human activity, e.g., the number of incoming boats at a given port^17–21^.

Simultaneously (particularly in cases of accidental transfers), we can expect human-mediated dispersal sources in areas of high human activity. Therefore, urban areas with higher frequencies of dispersal vectors may be associated with increased dispersal of invasive species, both incoming and outgoing. Such twofold effects of vector density have been integrated into models that explicitly consider the flow of dispersers^17,19–21^, while other models consider only one side of these effects. By comparing models with and without both effects in the same framework, we can identify qualitative differences between these two types of modeling strategies.

Management of invasive species requires a targeted approach for maximum effect. A number of studies using spatially explicit models have demonstrated that where the effort is initially targeted can have a profound effect on outcome^5,22–25^. Controls based on population biology have generally supported prioritization of the edges of a population as primary targets of initial control efforts^5,23,24,26^. On the other hand, studies focused on metapopulations often support prioritization of core populations that supply most of the new propagules^23,26,27^. Further, the optimal spatial arrangement of the control effort is considered to be highly context-dependent^5^. Therefore, we need to understand the mechanisms underlying these different expectations for improved control success.

In this study, we investigated spatially heterogeneous long-distance dispersal rates using a scatter colony model^28–31^ to derive a factor for determining how heterogeneous environments influence the early population dynamics of invasive species. We considered a finite-sized continuous area that is parametrized by two spatial functions, (1) intensities to attract dispersers of the invasive species (destination function) and (2) intensities to produce dispersers of the invasive species (source function). Those two spatial functions describe how spatial heterogeneity influences long-distance dispersal of the invasive species. In this study, we defined and investigated three types of models (1) destination-mediated-dispersal models (spatially heterogeneous destination function and uniform source function), (2) source-mediated-dispersal models (spatially uniform destination function and heterogeneous source function), and (3) full models of which destination and source functions are spatially heterogeneous. By comparing analytical investigations with spatially explicit stochastic models, we demonstrated how to predict early population dynamics for initial control success with considering spatial heterogeneity of dispersal.

## Results

### Heterogeneous destination function decelerates population growth

**Figure 1:**
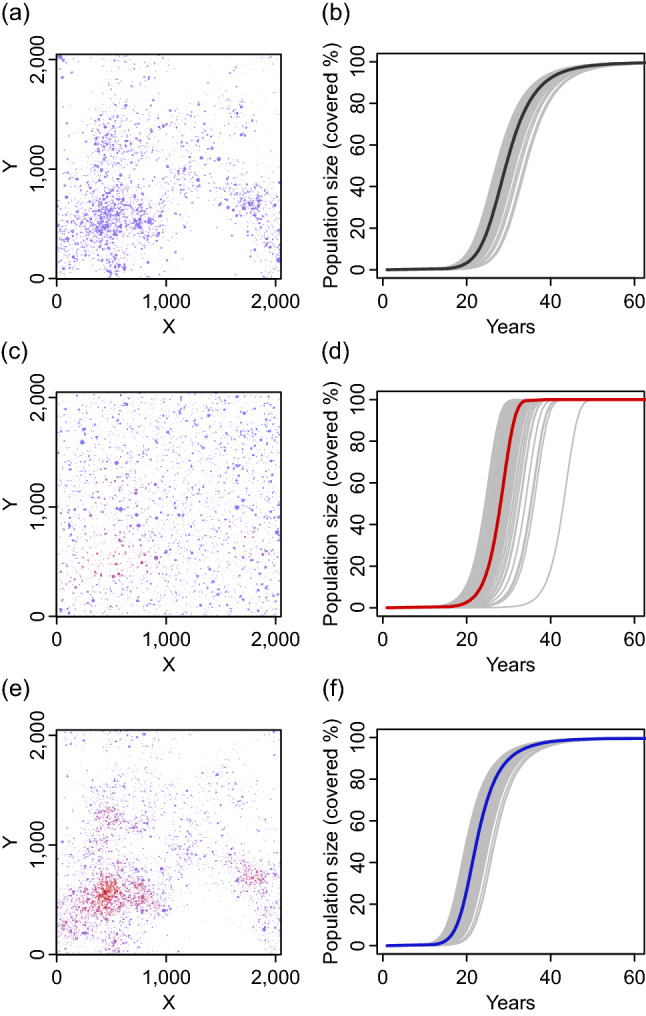
Spatial settings influence realized spatial distributions of colonies and their population dynamics: (**a**, **b**) destination-mediated-dispersal model, (**c**, **d**) source-mediated-dispersal model, and (**e**, **f**) source–destination-mediated-dispersal model. (**a**, **c**, **e**) Colored regions show colonies that cover 10% of the entire area (ages of these populations are 28, 35, and 18, respectively). Colors of these regions indicate colonies with low (blue) to high (red) dispersal frequencies. White to gray shades indicate the magnitude of the background intensity distribution (darker for larger intensities). (**b**, **d**, **f**) Thin gray curves indicate 100 independent time courses for proportions of areas occupied by colonies. Thick curves show the average time in which a population reaches a certain proportion. All realizations, including realizations for panels (**a**, **c**, and **e**), use the same background intensity distribution ($F_{h}^{{\tfrac{1}{3}}} = 1.40$).

**Figure 2:**
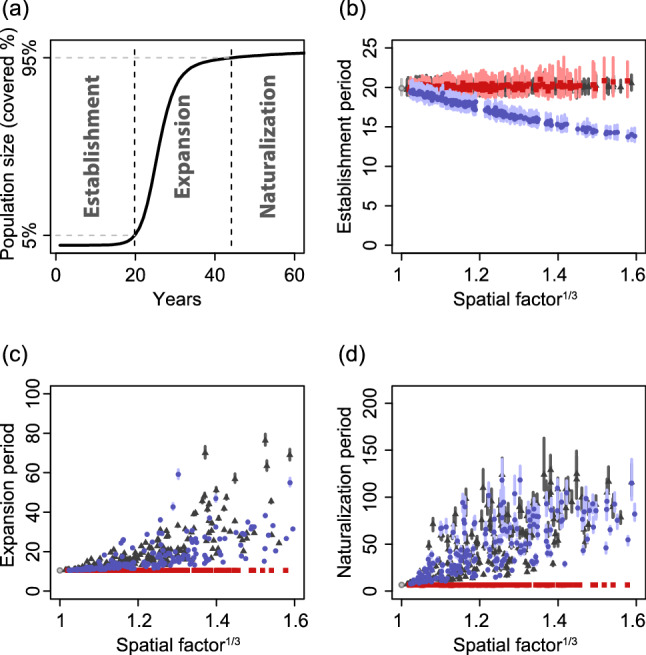
Effects of spatial heterogeneity of dispersal vectors on population dynamics as a function of model type. (**a**) Schematic describing three phases used; (i) establishment (0–5%, panel **b**), (ii) expansion (5–95%, panel **c**), and (iii) naturalization (95–100%, panel **d**). (**b**–**d**) Response periods of phases based on the spatial factor. Points and corresponding vertical lines represent medians and 25%–75% quantiles of 100 realizations, respectively. Black triangles, red squares, and blue circles correspond to destination-mediated-dispersal, source-mediated-dispersal, and source–destination-mediated-dispersal models, respectively. Black open circles and lines on the left point (spatial factor $F_{{\text{h}}} = 1$) correspond to a null model without any spatial heterogeneities.

When the destination function was spatially heterogeneous but the source function was homogeneous (i.e., destination-mediated-dispersal model), colonies aggregated (Fig. 1a) and the population initially expanded rapidly but decelerated in the later part of the time course (Fig. 1b). The lengths of the establishment phase of the destination-mediated-dispersal models were invariant of the spatial factor $F_{{\text{h}}}$ (Fig. 2b, black triangles). On the other hand, larger spatial factors tended to delay population growth within the expansion phase (Fig. 2c, black triangles). Covering the last 5% of area (the naturalization phase) took a longer period of time when the spatial factor was large (Fig. 2d, triangles); sometimes the naturalization phase was longer than the expansion phase. Therefore, with spatially heterogeneous attractiveness alone, spatial arrangements of the dispersal vector $h(x,y)$ do not affect the establishment of a population, but a large spatial factor value tends to delay population growth in the following period.

### Heterogeneous source function increase stochasticity of early dynamics

When the source function was spatially heterogeneous but the destination function was homogeneous (source-mediated-dispersal models), colonies scattered on the space (Fig. 1c) and the length of the early growth period varied greatly relative to those other types of models (Figs. 1d and 2b, red squares). The lengths of the establishment periods of the source-mediated-dispersal models responded positively to the spatial factor (Fig. 2b, red squares). Periods of subsequent phases (expansion and naturalization) of the source-mediated-dispersal models did not respond to the spatial factor (Fig. 2c,d, red squares). Therefore, spatially heterogeneous source function can induce large fluctuations in the establishment of a population, but has little effect on the later phases of establishment and expansion.

### Spatial heterogeneities accelerate early population growth in models that assume both source and destination functions vary spatially

In the full models, colonies aggregated in areas with high dispersal frequency because both attractiveness and dispersal frequency are proportional to the same function $h(x,y)$ (Fig. 1e). Unlike the two previous models, the lengths of the establishment phases decreased with an increase in the spatial factor (Figs. 1f and 2b, blue circles). In the full models, the periods of the establishment phases varied among realizations and were smaller than those of the source-mediated-dispersal models (Fig. 2b, blue circles). In the full model the establishment and expansion phases responded similarly to the destination-mediated-dispersal models. Larger values of the spatial factor resulted in longer expansion and naturalization phases (Fig. 2c,d, blue circles). Thus, a major characteristic of the full models is a rapid population growth in a heterogeneous landscape just after the invasion.

**Figure 3:**
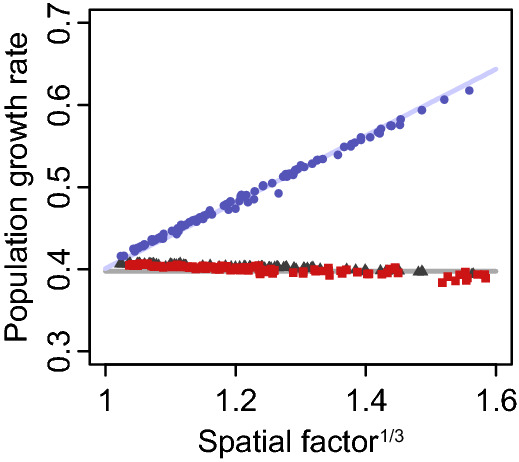
Asymptotic growth rates of full models increase with increases in the spatial factor, while other rates are invariant of the factor. Gray triangles, red squares, and blue circles indicate asymptotic growth rates estimated from realizations with source-mediated-dispersal, destination-mediated-dispersal, and source–destination-mediated-dispersal models, respectively. The open circle at a unit spatial factor represents a model without any spatial heterogeneities. Solid lines indicate theoretical growth rates with (blue) and without (gray) influence of spatial factors.

The estimated asymptotic growth rates from the destination-mediated-dispersal and source-mediated-dispersal models did not respond to spatial distribution of the dispersal vectors (Fig. 3, gray triangles and red squares). These estimated values did not differ from the growth rate at $F_{{\text{h}}} = 1$ (i.e., the asymptotic growth rate of the spatially homogeneous model). On the other hand, in the full models, large values of the spatial factors resulted in large values of estimated asymptotic growth rates (i.e., faster population growth after the initial introduction) (Fig. 3, blue circles). These estimated values correspond well with the theoretical expectations from Eq. (4) (Fig. 3, solid lines).

## Discussion

In this study, we considered two mechanisms of spatial heterogeneity involved in long-distance dispersal: spatially heterogeneous source functions and spatially heterogeneous destination functions. We analyzed these mechanisms to clarify how heterogeneous distributions of dispersal vectors influence regional population dynamics. Our results show that a combination of these mechanisms, not either of them in isolation, addresses spatial effects on the asymptotic growth rate in the initial success of establishing a population.

### Spatial heterogeneity and destination-mediated dispersal

When spatial heterogeneity in vector density controls the destinations of long-distance dispersers (i.e., a destination function) in isolation, our modeling suggests that once the population covers a large proportion of available area, there is a delay in population expansion. In a spatially heterogeneous destination, the population aggregates in areas of higher value, resulting in missed dispersal opportunities by re-establishing in areas already occupied by existing sub-populations. This scenario is applicable to introductions of a species to remote areas arrive from outside the focal area (e.g. from forestry plantations to urban areas).

### Spatial heterogeneity and source-mediated dispersal

When spatial heterogeneity of vector density controls the number of dispersers (i.e., a source function) only, the total number of dispersers produced from a population depends on the spatial arrangement of sub-populations. This was caused by a selection of population area in the analyses of the models. If sub-populations cover most of the area, the total number of dispersers will be very close to its expected value. Alternatively, if a population consists of a few small colonies, the total number of dispersers produced varies depending on the actual spatial distribution of the sub-populations. Therefore, spatial heterogeneity in the source function introduces uncertainty in the early phase of population dynamics, which diminishes as the population establishes and expands.

### Spatial heterogeneity of both source and destination functions

The most notable outcome from our three model settings is that spatial heterogeneity influences initial population growth only when the source and the destination functions are both heterogeneous and are spatially correlated (Supplemental Information SI 3). Source and destination correlation results in a higher initial rate of propagule production. On the other hand, after the population covers most of an area, its expansion slows because dispersers are rarely introduced to remaining open areas because of their very low values of the destination function. In this situation, instead of establishing in these open areas, most dispersers are transferred to already populated areas. Among three types of models investigated in this study, only the full models relate spatial heterogeneity with an asymptotic growth rate, which is a key determinant of both extinction probability and invasiveness. The spatial factor we derived here can be considered to be a measure of correlations between the source and the destination functions (without subtracting mean values). Therefore, the full model type is distinct from the others for its ability to determine the effects of long-distance dispersals in the population dynamics of an invading species, especially on the species’ responses to control strategies.

### Source and destination mediated dispersal and control of core populations

The spatial factor can be reduced by decreasing the dispersal frequency or attractiveness of an area with a high density of dispersal vectors. Therefore, if both source and destination functions influence dispersal success, our model suggests potential eradication success through simultaneous strategies that effectively weaken dispersals between core areas, where colonies tend to produce more propagules than other areas. Similar control strategies focusing on core populations have been suggested by metapopulation models^23,26,27^. These strategies have been applied in cases of invasive aquatic invertebrates and fish larvae transferred accidentally by ballast water^32,33^. On the accidental transfer of aquatic species by ballast water or attaching to boats, the number of boats in a port approximates both the inflow (destination function) and outflow (source function) of invader species’ propagules. In addition, using the common assumption of stationarity in the number of boats in each port^17,19,21,34^, the attractiveness and the dispersal frequency of each port also becomes stationary. This introduces time-invariant correlations between the attractiveness and the dispersal frequency of a sub-population. Our results suggest that this correlation between attractiveness and dispersal frequency introduced by a stationary density of boats points to the importance of core populations. Similar discussions may also be applicable to accidental transfers of terrestrial small organisms attaching to vehicles and machinery, which is an important pathway for spreading insect invaders^35–37^.

### Edge expansion and population growth

We emphasize that our models do not contradict the common rule of thumb that recommends prioritizing control at the edges of a population^5,23,24,26^. The asymptotic growth rates in our models are essentially the same formulation as a spatially homogeneous model, with a larger influence of contributions from short-distance dispersal than those of long-distance dispersal. Thus, we expect a larger influence from the proportional change of the short-distance dispersal speed (i.e., expansion speed at edges) than from the frequency of long-distance dispersal^31^. Note that influences from short- and long-distance dispersals on the asymptotic growth rate in our models are multiplicative rather than additive. Therefore, the measures of the additive effects of parameters can indicate a stronger influence from long-distance than short-distance dispersal^38,39^, especially when short-distance dispersal is weak.

## Conclusion

Our modeling emphasizes the importance of the basic assumptions regarding how propagules of invader species interact with vectors mediating long-distance dispersal. Different types of interactions can lead to qualitatively different conclusions when considering population dynamics produced by spatially heterogeneous distributions of vectors. These models and results bridge the gap between discrepancies between strategies, helping to drive decisions to focus on edge *versus* core populations (a result of using two different stems of modeling frameworks). From the present result, we conclude that clamped spatial distribution of a human population can accelerate invasive species’ range expansion after establishment, if the human-population density influences both starting and ending locations of the invader species’ dispersal (e.g., accidental transfer by vehicles). This outcome will facilitate a better mechanistic understanding of how different types of vectors influence invasive species’ spread, and support the development of robust models to predict their future expansions.

## Methods and models

### Target species and basic assumptions

**Figure 4:**
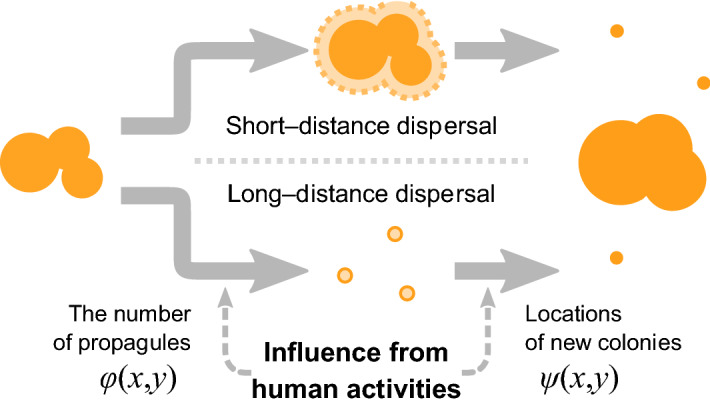
A schematic of short- and long-distance dispersal modes. Human activities may influence the long-distance dispersal by: (1) changing the number of propagules starting the long-distance dispersal ($R \cdot \varphi (x,y)$), and (2) introducing biases in their spatial locations ($\psi (x,y)$). By compositing these short- and long-distance dispersals, we predict population establishment at the next time step.

We developed and analyzed spatially explicit models that describe range expansion of an invader species’ population that consists of many sub-populations. Invasive species often expand their range by stratified dispersal using human-mediated long-distance dispersal in addition to local expansion of sub-populations^20^. Extending a model rigorously analyzed by Takahashi et al.^31^, which explicitly involves (1) short-distance dispersal that expands the area of current sub-populations, and (2) long-distance dispersal that establishes new sub-populations beyond existing sub-populations (Fig. 4), we consider spatially inhomogeneous factors that influence on the long-distance dispersal (e.g., vectors’ distribution). The parameters and functions used in these models are listed in Table 1.

**Table 1:** Symbols and their default values.

Symbol	Description	Unit	Default value
$x$, $y$	Standardized spatial coordinate	$L$	–
$\rho_{t} (x,y)$	Area covered by a population at year $t$	1	–
$S$_,_ $\left| S \right|$	Study area ($\left| S \right|$ is the size of the study area)	–	–
$g$	Maximum length of the short-distance dispersal (a length unit $L$ is selected to keep this parameter to be 1)	$L \cdot {\text{year}}^{ - 1}$	1
$R$	Average long-distance dispersal frequency per year per unit area	$L^{ - 2} \cdot {\text{year}}^{ - 1}$	0.01
$h(x,y)$	Spatial distribution of the human-mediated dispersal vectors (standardized to be 1 in total)	1	–
$\varphi (x,y)$	Frequency of starting the long-distance dispersal from a population at $(x,y)$ (source function)	1	–
$\psi (x,y)$	Likelihood of a sub-population establishment at $(x,y)$ (destination function)	$L^{ - 2}$	–
$F$	Overall magnitude of effects human activities on growth rate (spatial factor)	1	–

We estimated the population size of the invader species as the area covered by any of these sub-populations in the study area. This simple measure of the population size was based on our assumption that the inside of a sub-population is homogeneous and these sub-populations vary only in their positions, sizes, and shapes. This assumption may oversimplify spatial architectures of the sub-populations, because even clonal colonies of perennial plants often have concentric structures that can influence reproductive output^40^; but this simplification is applicable when the reproductive rate of the species is high enough to reach a constant carrying capacity quickly.

### Short- and long-distance dispersals

We considered stratified dispersals of the invader species. Note that we explicitly considered invader species’ dispersal but their vectors’ movement (e.g., human traffics) was included only implicitly. The short-distance dispersal expands the species’ range only by a constant velocity^7^. Therefore, we modeled the short-distance dispersal as radial expansion of these sub-populations with a constant speed *g*. Meanwhile, long-distance dispersal introduces new sub-populations into the population out of its parent sub-population. Empirical observations showed that long-distance dispersal mediated by human activities introduces a new sub-population to an area at a long distance from its source population, e.g., vehicles can move plant seeds for more than hundreds of kilometers^41^. Long-distance dispersal diminishes the influence of the source location on the destination of a dispersal event, so as a simplifying approximation we assumed that the destination of the long-distance dispersal is independent of the source location of a sub-population.

The assumption of source location independence of the dispersal destination allows us to describe a process of long-distance dispersal by two functions on $S$: (1) a function $\varphi (x,y)$ describing spatial variation in disperser production rates, and (2) a function $\psi (x,y)$ describing the probability that a coordinate $(x,y)$ is selected as a destination of the disperser. Following the notation of Jongejans et al.^18^, we call $\varphi (x,y)$ and $\psi (x,y)$ the source and destination functions, respectively. Note that we define the spatial average of the source function to be one (i.e., ${{\int_{S} {\varphi (x,y)\,dxdy} } \mathord{\left/ {\vphantom {{\int_{S} {\varphi (x,y)\,dxdy} } {|S|}}} \right. \kern-\nulldelimiterspace} {|S|}} = 1$). We define *R* as the regional average of the disperser production rate per unit area (a spatially homogeneous component of the disperser production rate). Using this formulation, we calculate an expected disperser production rate of a given area by integrating $R\varphi (x,y)$ over the area. The spatial integration of the destination function over the study area is one because we assume the population of the invader species will reach full carrying capacity.

We have defined a population of the invader species as a spatial union of all sub-populations in the study area because we assume sub-populations are homogeneous. Within the study area, $\rho_{t} (x,y)$ is 1 if the coordinates $(x,y)$ are within at least one of the sub-populations of the invader species at time $t$ (and otherwise 0). The integration of $R\varphi (x,y)$ is the expected disperser production rate for a given $\rho_{t} (x,y)$, and the integration $R\int_{S} {\rho_{t} (x,y)\varphi (x,y)\,dxdy}$ is the expected total production of dispersers from a population in the *t*-th time step. Thus, assuming that the total number of dispersers that start long-distance dispersal at time $t$ (denoted by $n_{{{\text{d,}}t}}$) follow a Poisson distribution, we can write a probability that the population produces *k* dispersers in the *t*-th time, Eq. (1).

1 $$ \Pr [n_{{{\text{d}},t}} = k] = \frac{{\lambda^{k} e^{ - \lambda } }}{k!}{, }\lambda = R\int_{S} {\rho_{t} (x,y)\varphi (x,y)\,dxdy} . $$

The destination of the disperser is determined by the destination function $\psi (x,y)$, which determines probabilities of ending a dispersal event at $(x,y)$, which results in a new sub-population being established at that location. In total, the spatial distribution of new sub-populations introduced by long-distance dispersal follows a Poisson point process of which intensities are given as $\psi (x,y)R\int_{S} {\rho_{t} (x,y)\varphi (x,y)\,dxdy}$.

### Three model types

We compared three different types of models by varying interactions between the species’ long-distance dispersal and human activities. These included: (1) a source-mediated-dispersal model assuming that the source function $\varphi (x,y)$ varies spatially by human activity while the destination function $\psi (x,y)$ is uniform, (2) a destination-mediated-dispersal model assuming that the destination function varies spatially while the source function is uniform, and (3) a full model assuming that both source and destination functions vary spatially.

Let $h(x,y)$ be a function representing intensity of human activities at coordinates $(x,y)$. Without loss of generality, we can assume that the function $h(x,y)$ satisfies $\int_{S} {h(x,y)\,dxdy} = 1$, the total intensity over the study area is scaled to one. In the source-mediated-dispersal model, the source function is proportional to the human-activity intensity, i.e., $\varphi (x,y) = |S| \cdot h(x,y)$, and the destination function $\psi (x,y)$ is uniformly equal to ${1 \mathord{\left/ {\vphantom {1 {|S|}}} \right. \kern-\nulldelimiterspace} {|S|}}$. On the contrary, the source function of the destination-mediated-dispersal model is uniform and the destination function is $\psi (x,y) = h(x,y)$. The full model is a combination of the source- and destination-mediated-dispersal models in which both source and destination functions vary with area. In this study, we assume that a single factor determines both the source and destination functions, i.e., $\varphi (x,y) = \left| S \right| \cdot h(x,y)$ and $\psi (x,y) = h(x,y)$ (Table 2).

**Table 2:** Definitions of model types.

Model type	Source function $\varphi (x,y)$	Destination function $\psi (x,y)$
Source-mediated dispersal model	$|S| \cdot h(x,y)$	${1 \mathord{\left/ {\vphantom {1 {|S|}}} \right. \kern-\nulldelimiterspace} {|S|}}$ (uniform)
Destination-mediated dispersal model	1 (uniform)	$h(x,y)$
Full model	$|S| \cdot h(x,y)$	$h(x,y)$

### Asymptotic growth rate of a population

Spatial dimension introduces complexity, though rigorous mathematical analysis is still viable for a small population with few small sub-populations. This situation may arise with an accidentally transferred population. Here, we consider infinitesimally small populations to be rare for invasive species and derive an asymptotic value of the growth rate to the size of the area inhabited by the population.

Each sub-population includes age, so we incorporated age-structured population dynamics^29,31^ into the model, described by the differential equation,

2 $$ \frac{\partial n(a,t)}{{\partial t}} + \frac{\partial n(a,t)}{{\partial a}} = 0, $$

where $n(a,t)$ represents the frequency of sub-populations with age *a* at time *t*. Note that Eq. (2) assumes no extinction of sub-populations. The equation has two boundary conditions: (1) $n(a,0)$ represents an age distribution of the initial population, and (2) $n(0,t)$ represents the number of new sub-populations (i.e., age 0 sub-populations) introduced by the long-distance dispersal at time *t*.

To determine the number of new sub-populations, we need to determine how many long-distance dispersers will emerge from a given population by including spatial heterogeneities. Recall that a sub-population expands outward by a constant speed *g*. Therefore, if we ignore overlaps among sub-populations each sub-population keeps a circular shape of radius proportional to age. In addition, if a sub-population is young, i.e., its size is small, we can regard the value of the source function $\varphi (x,y)$ inside the sub-population as uniform. Let $(x_{i} ,y_{i} )$ and $a_{i}$ be the position of the center and age of the $i$-th sub-population, respectively. With the above approximations, we can simply derive the expected number of long-distance dispersers that start dispersal from the $i$-th sub-population as $\pi R \cdot (ga_{i} )^{2} \varphi (x_{i} ,y_{i} )$.

On the other hand, existing sub-populations also originate from long-distance dispersal. Therefore, a position of the sub-population also follows the destination function $\psi (x,y)$. Building on the expected number of new sub-populations we described at the last paragraph, we calculate an average over the study area to calculate a mean-field approximation of the number of long-distance dispersers from an age *a* sub-population as $\pi R\int_{S} {\psi (x,y)(ga)^{2} \varphi (x,y)\,dxdy}$.

We assume that a population consists of a few small sub-populations in this formulation and a disperser will always establish outside existing sub-populations. Therefore, the total number of a new sub-population (i.e., $n(0,t)$) is a summation of new sub-populations produced by each of the existing sub-populations,

3 $$ \begin{gathered} n(0,t) = \pi R\int_{0}^{t} {n(a,t)\left[ {\int_{S} {\psi (x,y)(ga)^{2} \varphi (x,y)\,dxdy} } \right]\,da} \\ = \pi R\left( {\int_{S} {\psi (x,y)\varphi (x,y)\,dxdy} } \right)\int_{0}^{t} {(ga)^{2} n(a,t)\,da} . \\ \end{gathered} $$

With Eq. (3), the asymptotic growth rate of the Eq. (2) can be calculated as follows^29,31^,

4 $$ \left( {2\pi Rg^{2} \int_{S} {\psi (x,y)\varphi (x,y)\,dxdy} } \right)^{1/3} . $$

The asymptotic growth rate indicates that the integration $\int_{S} {\psi (x,y)\varphi (x,y)\,dxdy}$ describes the influence of the source and the destination functions in the early phases of population growth. Therefore, hereafter we call $\int_{S} {\psi (x,y)\varphi (x,y)\,dxdy}$ a spatial factor $F_{{\text{h}}}$ of the long-distance dispersal. Note that the spatial factor reduces to 1 for both source- and destination-mediated dispersal models. For the full models of which source and destination functions are $\varphi (x,y) = \left| S \right|h(x,y)$ and $\psi (x,y) = h(x,y)$, respectively, we can reduce the spatial factor $F_{{\text{h}}}$ to $\int_{S} {\left( {\sqrt {\left| S \right|} h(x,y)} \right)^{2} dxdy}$, equivalent to Simpsons’ diversity index.

### Numerical analysis

We evaluated the effects of the dispersal vector on distribution with an individual-based approach that describes colonies in a population as groups of individuals within a circular shape of various sizes (Fig. 1a,c,e for typical model outputs, see supplemental information SI 1 for detailed settings). To evaluate the effect of spatial heterogeneity on population dynamics, for each model type we generated 100 of $h(x,y)$ randomly (see shaded area of Fig. 1a,c,e, and SI 2 for the algorithm used) and ran 100 independent realizations for each $h(x,y)$. For each realization, we split the time course into three phases: establishment, expansion, and naturalization. The phases were based on the proportion of area inhabited by the population (Fig. 2a; less than 5%, 5% to 95%, and 95% to complete occupation of the total area, respectively), and measured the length of each phase. We linearly interpolated the time course based on area covered for each phase.

Using the same set of realized time courses, we estimated the asymptotic growth rate as the peak of a distribution of the logarithmic value of the instantaneous growth rate, defined as a difference of logarithmic values of covered area that are adjacent in a time course. We excluded periods that a population covers less than 1% or more than 50% of the total area to avoid strong demographic stochasticity of initial dynamics and a deceleration phase of S-shaped growth, respectively. We gathered these logarithmic values of instantaneous growth rates from 100 realizations with the same $h(x,y)$ and dispersal type, then estimated the density distribution using Gaussian kernel estimation. Finally, we determined the maximum point of the estimated density distribution as the estimated asymptotic growth rate.

## Supplementary Information

Supplementary Information.
