# Supplementary material for: Spatial heterogeneities of human-mediated dispersal vectors accelerate the range expansion of invaders with source–destination-mediated dispersal
Source: Sci Rep. 2020 Dec 8;10:21410. doi: 10.1038/s41598-020-78633-3 (PMC7722924; doi:10.1038/s41598-020-78633-3)
Supplement: Supplementary file 1 — Supplementary Information. [file 41598_2020_78633_MOESM1_ESM.pdf]

# Spatial heterogeneities of human-mediated dispersal vectors accelerate the range expansion of invaders with source-destination-mediated dispersal

Daisuke Takahashi, Young-Seuk Park

## SI 1. Numerical settings

Without loss of generality, in the numerical models, we can regard the annual colony expansion $g$ as a unit length by scaling all other variables and parameters, and in the present study, we set the size of the focal area as $l = 2,048$, i.e., 2,048 times larger than the annual colony expansion. Then we approximate $\rho_t(x, y)$ by rasterizing a population into a $2,048 \times 2,048$ square grid for each time step. A large area size results in slower population dynamics with less temporal fluctuations; however, note that the asymptotic–growth rate and the spatial factor are non-dimensional and invariant of the area size and the spatial unit.

If otherwise specified, we independently generate the vector distribution $h(x, y)$ with randomly selected parameters from $\alpha \in [1,5]$ and $\beta \in [-5,-1]$ before each realization, rasterize the distribution into a $512 \times 512$ square grid, and expand its size by four. The resolution of the grid is lower than the population grid described above (i.e., a grid cell of the vector distribution covers a 16-times bigger area than that of a grid cell of the population grid), due to the large computational demand for determining colony locations. This resolution reduction results in loss of the fine-scale spatial variance of background intensity distribution, which may introduce a bias in the resultant population dynamics. However, for several selected parameter combinations, we qualitatively got the same time courses with and without resolution reduction (not included). Therefore, we consider that resolution reduction does not affect our conclusion.

We implemented colony removal in the present models as follows; at the end of each time step, we (1) rasterize the whole population on a grid again, (2) break the rasterized grid into patches of connected cells that are covered by colonies, (3) count an area size of each patch, and (4) remove all colonies on a patch if its size exceeds the given threshold representing the size needed to detect a colony.

## SI 2. Spatial distribution of human-mediated dispersal vectors

To extract the general properties of the dynamics, we need a randomly generated vector distribution $h(x,y) \geq 0$. To get such random distributions, on the present study, we adopted an algorithm that have been proposed as a model of ecological spatial structures [1–3] that is fundamentally the same algorithm as a classic approximation of fractional Brownian motions.

The algorithm realizes a spatial distribution of intensities, namely $M(x,y)$, by applying inverse discrete Fourier transform (IDFT) on randomized 2-dimensional phases with given power-spectral-density distribution. The resultant $M(x,y)$ will be isotropic surface with a given autocorrelation structure [3]. Note that we need to keep antisymmetric structure of randomized phases to get real-valued surface [1]. The algorithm has a parameter $\beta < 0$, which determines the power of the power-spectral-density distribution. Informally speaking, small values of $\beta$ result in smooth spatial distributions of $M(x,y)$.

We considered that the values of generated $M(x,y)$ are background (unobservable) factors determining the vector distribution $h(x,y)$. By assuming a power-law response of the vector density to the background factors, we defined the non-negative vector distribution $h(x,y)$ as,

$$h(x,y) = c\left(M(x,y) - \min\left(M(x,y)\right)\right)^{\alpha}. \qquad \text{S1}$$

The parameter $\alpha$ is a response power of which high values exaggerate spatial distribution of the background factors. We do not need the subtraction of the minimum $M(x,y)$ if we control a DC component of the power spectral-density distribution. To focus on spatial distributions rather than the total amount of vectors in the area, we kept an integration $\iint h(x,y)dxdy$ over the area to be 1 by a factor $c$. We note that the resultant $h(x,y)$ may no longer have a power-law power-spectral-density distribution. Fig. S1 shows eight examples of generated vector distributions $h(x,y)$ that are sorted by corresponding values of $F_{\mathrm{h}}^{1/3}$ defined in the main text.

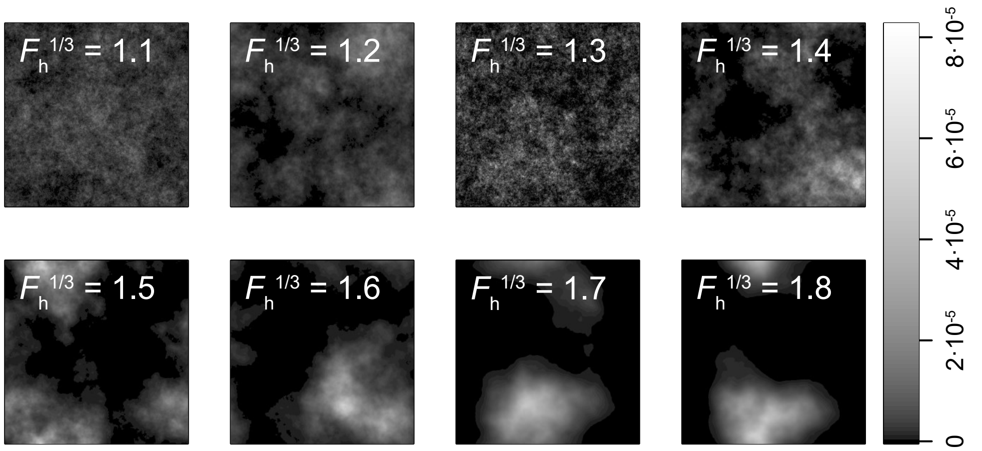


Figure S1: Values of $h(x,y)$ generated from Equation S1 with corresponding values of $F_{\mathrm{h}}^{1/3}$.

## SI 3. Independent source and destination functions and estimated asymptotic growth rate

In the main text, we assume that the source and destination functions are (1) spatially heterogeneous but derived from an identical vector distribution or (2) only one of them is spatially heterogeneous. Here, we relax this assumption for more generality of our argument.

We determined two vector distributions $h_s(x,y)$ and $h_d(x,y)$ independently using the algorithm described at SI 2. Parameters $\alpha$ and $\beta$ are independently chosen from $[1,5]$ and $[-5,-2]$, respectively. Then we determine the source function $\varphi(x,y)$ and the destination function $\psi(x,y)$ as $|S|h_s(x,y)$ and $h_d(x,y)$, respectively, and we realized a time course with the same parameter values as in the main text.

We iterated above realizations for 100 times, each time we determined vector distributions independently, and estimate the asymptotic growth rate of each time course (Fig. S2). Same as we described in the main text, estimated asymptotic growth rate is almost identical to theoretical values of the asymptotic growth rate $\left(2\pi Rg^2 F_h\right)^{1/3}$, where $F_h = \int_S \left(\sqrt{|S|}h_s(x,y)h_d(x,y)\right)^2 dxdy$, showing strong relationships between the asymptotic growth rate and the spatial factor we are discussing.

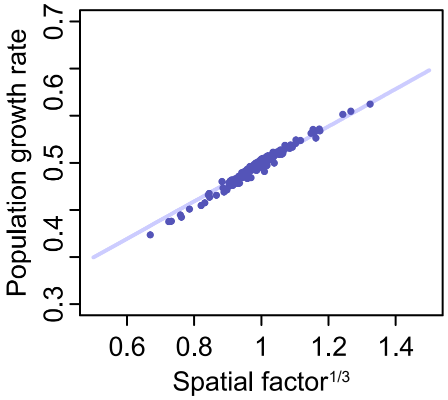


Figure S2: Estimated asymptotic growth rates (blue circles) are almost linear function of cubic root of the spatial factor $F_h$. A blue line indicates theoretical values. The source and the destination functions are determined by using independently generated vector distributions. Note that the spatial factor can be below 1 because the source function $\varphi(x,y)$ is not equal to $|S|\psi(x,y)$ as in the source-destination-mediated-dispersal models in the main text.

## References


1. Chipperfield, J. D., Dytham, C. & Hovestadt, T. An Updated Algorithm for the Generation of Neutral Landscapes by Spectral Synthesis. *PLOS ONE* **6**, e17040 (2011).
2. Keitt, T. H. Spectral representation of neutral landscapes. *Landsc. Ecol.* **15**, 479–494 (2000).
3. Lennon, J. J. Red-shifts and red herrings in geographical ecology. *Ecography* **23**, 101–113 (2000).
